# Supplementary material for: Revealing the role of interfacial water and key intermediates at ruthenium surfaces in the alkaline hydrogen evolution reaction
Source: Nat Commun. 2023 Aug 30;14:5289. doi: 10.1038/s41467-023-41030-1 (PMC10468501; doi:10.1038/s41467-023-41030-1)
Supplement: Supplementary file 1 — Supplementary Information [file 41467_2023_41030_MOESM1_ESM.pdf]

Supplementary Information

Revealing the role of interfacial water and key intermediates at ruthenium surfaces in the alkaline hydrogen evolution reaction

Xing Chen,[1,#] Xiao-Ting Wang,[1,#] Jia-Bo Le,[2,#] Shu-Min Li,[1] Xue Wang,[2] Yu-Jin Zhang,[1] Petar Radjenovic,[1] Yu Zhao,[1] Yao-Hui Wang,[1] Xiu-Mei Lin,[1,3]* Jin-Chao Dong,[1,4]* and Jian-Feng Li[1,4]*

[1]College of Energy, College of Chemistry and Chemical Engineering, College of Materials, State Key Laboratory of Physical Chemistry of Solid Surfaces, iChEM, Xiamen University, Xiamen, 361005, China

[2]Key Laboratory of Advanced Fuel Cells and Electrolyzers Technology of Zhejiang Province, Ningbo Institute of Materials Technology and Engineering, Chinese Academy of Sciences Ningbo 315201, China

[3]Department of Chemistry and Environment Science, Fujian Province University Key Laboratory of Analytical Science, Minnan Normal University, Zhangzhou 363000, China

[4]Innovation Laboratory for Sciences and Technologies of Energy Materials of Fujian Province (IKKEM), Xiamen 361005, China

[#]These authors contributed equally: Xing Chen, Xiao-Ting Wang, and Jia-Bo Le.

*e-mail: xiu-mei.lin@xmu.edu.cn; jcdong@xmu.edu.cn; Li@xmu.edu.cn

This file includes:

Mass formula

Supplementary Figures 1-16

Supplementary Tables 1-4

Supplementary References

Mass formula

A deuterium isotopic substitution measurement on the Au@Ru surface in 0.1 M NaOH saturated with Ar was carried out, as shown in Figure 2c. We found that the peaks at around 1827 cm^{-1} and 1950 cm^{-1} in H_2O shift to lower wavenumbers at around 1328 cm^{-1} and 1402 cm^{-1} in D_2O. Thus, these two peaks should be correlated with the "H" atom and were attributed to the stretching vibrations of Ru-H.

For *H species, the related Raman frequency downward shift ratio (γ) in the D_2O experiment can be approximately analyzed by following the mass formula:

$$\begin{aligned}\gamma &= \nu(\mathrm{RuD}) / \nu(\mathrm{RuH}) \\ &= \frac{\sqrt{\mathrm{m}(Ru) + \mathrm{m}(D)}}{\sqrt{\mathrm{m}(Ru) * \mathrm{m}(D)}} / \frac{\sqrt{m(Ru) + m(H)}}{\sqrt{m(Ru) * m(H)}} \\ &= \frac{\sqrt{101 + 2}}{\sqrt{101 * 2}} / \frac{\sqrt{101 + 1}}{\sqrt{101 * 1}} \\ &= 71.1\%\end{aligned}$$

Supplementary Figures

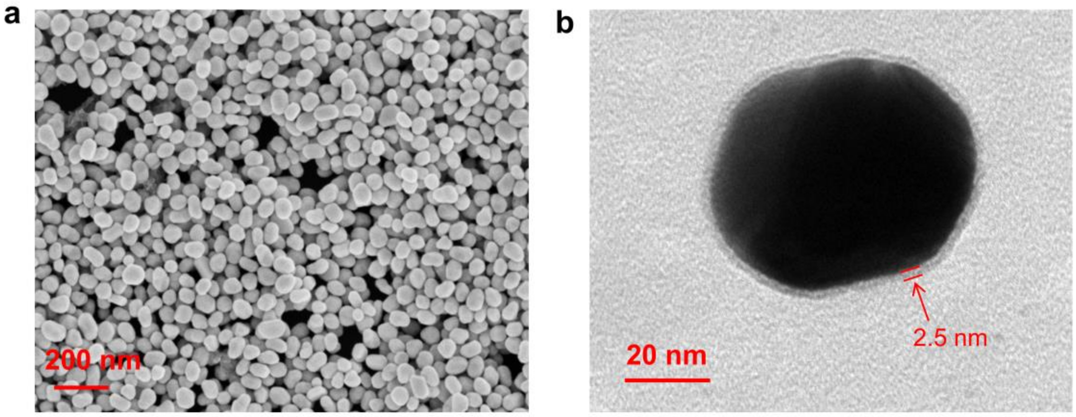

Supplementary Figure 1 | Electron microscopy characterization of 55 nm Au@2.5 nm Ru core-shell NPs. (a) Scanning electron microscopy (SEM) image, and (**b**) Transmission electron microscopy (TEM) image of the 55 nm Au@2.5 nm Ru nanoparticles (NPs).

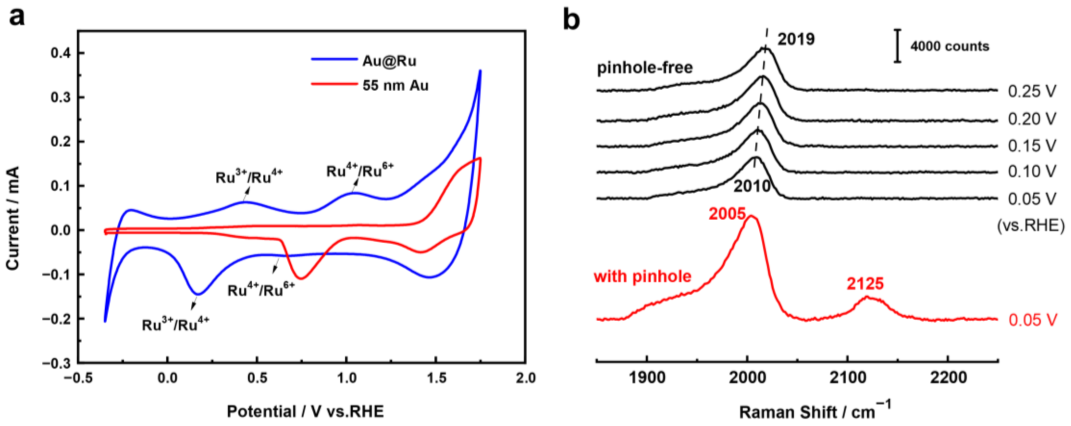

Supplementary Figure 2 | The pinhole test of Au@Ru NPs. (a) A comparison of cyclic voltammogram curves of 55 nm Au NPs (red) and 55 nm Au@2.5 nm Ru NPs (blue) in a solution of 0.1M $NaClO_4$. **(b)** Core-shell nanoparticle-enhanced Raman spectroscopy of CO adsorbed on 55 nm Au@0.7 nm Ru NPs (red, with pinholes) and 55 nm Au@2.5 nm Ru NPs (black, pinhole-free) in a solution of 0.1 M $NaClO_4$ saturated by CO.

The CV curve of 55 nm Au NPs (a, red) shows two Au reduction peaks at around 0.8 and 1.4 V[1-4]. The CV curve of 55 nm Au@2.5 nm Ru NPs (a, blue) shows two pairs of redox peaks at around 0.17/0.43 V and 0.67/1.04 V attributed to the Ru(+3)/Ru(+4) and Ru(+4)/Ru(+6)[4-6] whereas no Au reduction peaks appear, which indicates that the NPs are pinhole-free. We have also carried out new Raman measurements to further confirm the pinhole-free structure of the 55 nm Au@2.5 nm Ru NPs using CO as a probe molecule. The Raman spectrum of CO adsorbed on 55 nm Au@0.7 nm Ru NPs (b, red) at 0.05 V shows two Raman bands at around 2005 cm^{-1} and 2125 cm^{-1}. They are ascribed to $\delta_{CO(top)}$ on Ru[7,8] and $\delta_{CO(top)}$ on Au[9] respectively. The Ru shell of 55 nm Au@0.7 nm Ru NPs is filled with pinholes, which results in the probed CO molecule coming into contact with the Au core, the Raman band of $\delta_{CO(top)}$ on Au, therefore, can be observed. Nevertheless, the Raman spectrum of CO adsorbed on 55 nm Au@2.5 nm Ru NPs (b, black) shows only one Raman band of $\delta_{CO(top)}$ on Ru at around 2010 cm^{-1} at –0.05 V, and it blueshifts to around 2019 cm^{-1} at 0.25 V due to the Stark-tuning effect of the electrode. The absence of the Raman band of $\delta_{CO(top)}$ on Au again demonstrates the pinhole-free structure of the used 55 nm Au@2.5 nm Ru NPs.

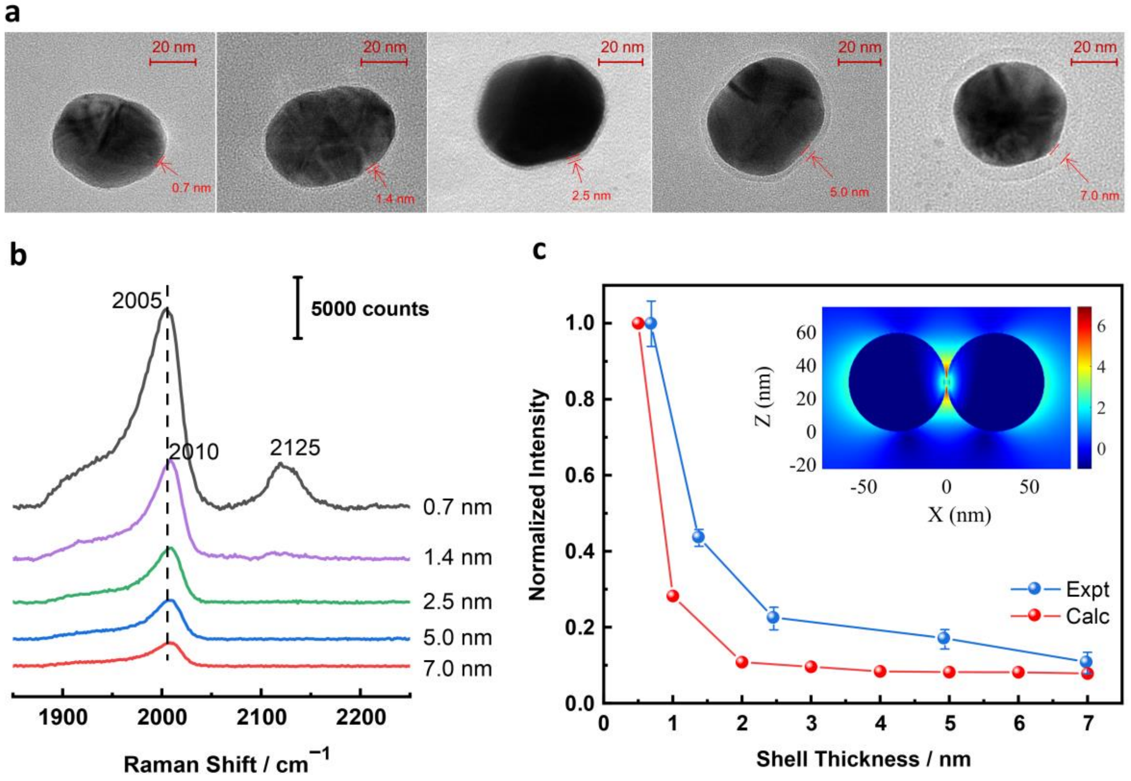

Supplementary Figure 3 | (a) TEM images of the Au@Ru NPs with different Ru shell thicknesses (0.7, 1.4, 2.5, 5.0, and 7.0 nm from the left to the right). **(b)** Corresponding enhanced Raman spectra of CO adsorbed on different thicknesses Ru shell Au@Ru NPs shown in (a) in a 0.1 M $NaClO_4$ solution saturated with CO at 0.05 V. **(c)** The shell thickness dependence of the normalized enhanced Raman spectra intensity of CO adsorbed on different Ru shell

thicknesses Au@Ru NPs (blue line) and the corresponding 3D-FDTD calculation result (red line). Inset is FDTD simulation of the electric field distribution on the surface of 55 nm Au@2.5 nm Ru dimer.

The intensity of the $\upsilon_{CO(top)}$ decreases dramatically as the interparticle distance controlled by the Ru shell thickness increases due to the decay of the plasmon coupling efficiency between particles. To make it more obvious, the intensity of the $\upsilon_{CO(top)}$ Raman band against Ru shell thickness was plotted (c, blue) and simulated by 3D-Finite-difference time-domain (3D-FDTD) (c, red). Our 3D-FDTD calculations prove that the corresponding highest enhancement of the Raman signal is 7×10^6 for the 2.5 nm shell. The strong enhancement effect of the electric field significantly amplifies the Raman signal of the species in our systems. They are therefore detected[10]. Additionally, it can be observed in (b) that the Raman band of $\upsilon_{CO(top)}$ at 55 nm Au@ 0.7 nm Ru is at 2005 cm^{-1}, which redshifts by 5 cm^{-1} on 55 nm Au@1.4 nm Ru and keeps consistent with increasing of Ru shell thickness till 7.0 nm. This indicates that the interaction between the CO and the Ru surfaces is influenced by the Au core only when the Ru thickness is thinner than 1.4 nm. Namely, the Au@Ru NPs are pinhole-free when the Ru thickness is thicker than 1.4 nm. Both experimental and theoretical results show the exponential decrease of the Raman intensity with increasing the shell thickness. Synergistically considering the pinhole-free structure and the Ru shell thickness-enhancement effect relationship, we selected the 55 nm Au@2.5 nm Ru NPs for our HER investigation.

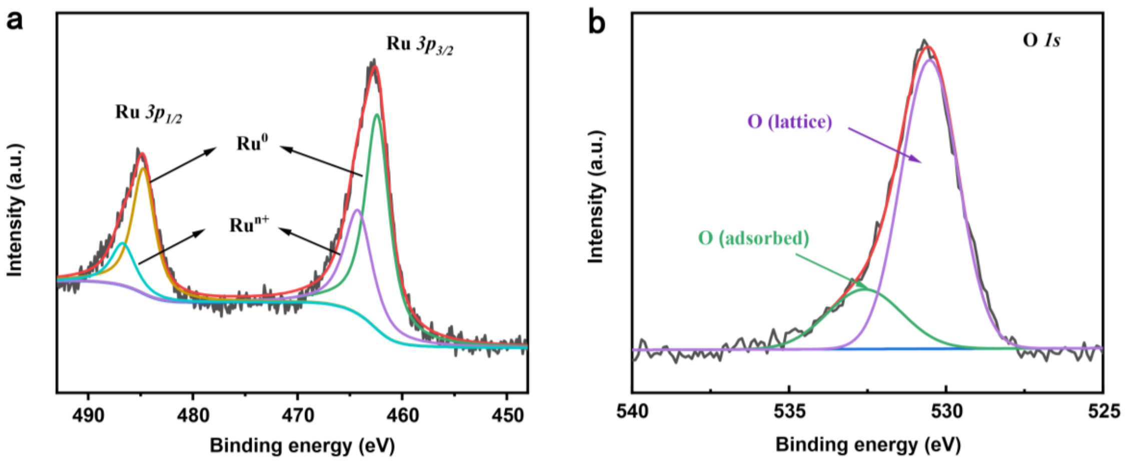

Supplementary Figure 4 | XPS characterization of Au@Ru NPs. XPS spectra of the Ru *3p* electrons **(a)** and O *1s* electrons **(b)** of Au@Ru nanocatalysts.

The XPS spectrum of the Ru *3p* electrons in (a) shows two characteristic peaks around 484.7 eV and 462.3 eV, corresponding to metallic Ru(0), and the other two peaks around 486.6 eV and 464.2 eV correspond to oxidized Ru(n+)[11]. And the O *1s* XPS spectrum in (b) can be assigned to lattice oxygen, absorbed hydroxide, and water (OH^-/H_2O) located at 530.4 and 532.5 eV, respectively[12,13]. The XPS analysis demonstrates the oxidation of the most out-layer of the Ru shell under atmospheric conditions. Therefore, different Ru valences are expected to be present on the Au@Ru NPs.

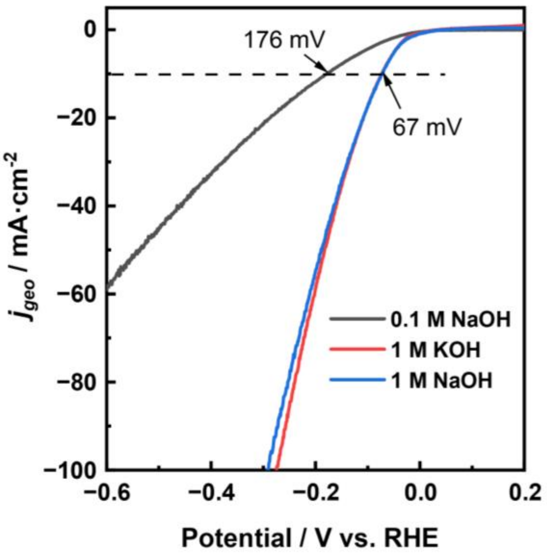

Supplementary Figure 5 | HER polarization curve at 55 nm Au@2.5 nm Ru surfaces in Ar-saturated 0.1 M NaOH (black), 1.0 M KOH (red), and 1.0 M NaOH (blue), 5 mV/s scanning rate and 1,600 r.p.m..

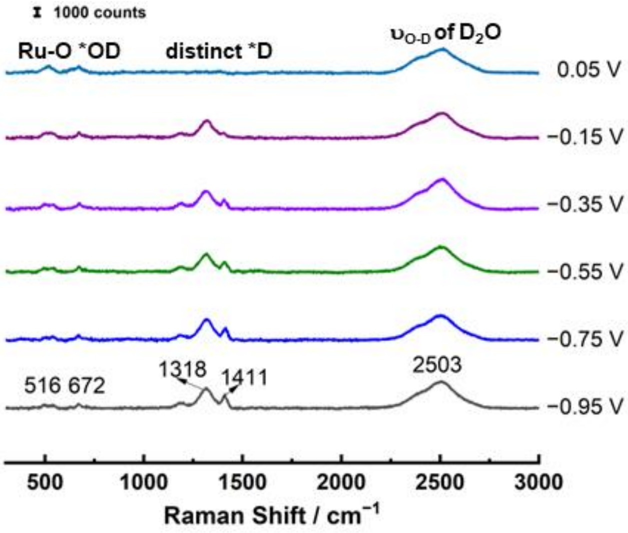

Supplementary Figure 6 | Deuterium isotopic substitution experiment of the HER catalyzed by 55 nm Au@2.5 nm Ru NPs in a D_2-saturated 0.1 M NaOD solution.

Generally, the changing trend of the Raman spectra (appearing or disappearing of the bands, changing in position and intensity of the existing bands) in the D_2O experiment is similar to that in H_2O. For example, in the isotope experiment, we also observed the Raman vibration signals of Ru-O (516 cm^{-1}), Ru-OD (672 cm^{-1}), two distinct D (1318 cm^{-1}, 1411 cm^{-1}), and interfacial water D_2O (2503 cm^{-1}) on Ru at −0.95V. The shifts of these vibrational frequencies accord with the expected shifts from the mass conversion of the formula (γ=71.1%) (as calculated, see mass formula section of Supplementary Information) and previous reports in the literature. With the potential shifts positively, the vibrational frequencies of Ru-O and Ru-OD are unchanged, and the intensity of *D on the high-valence Ru (1411 cm^{-1}) gradually decreases whereas the frequency of *D on zero-valence Ru (1318 cm^{-1}) redshifts. Our isotope experiment further verifies the phenomenon observed in the H_2O experiment.

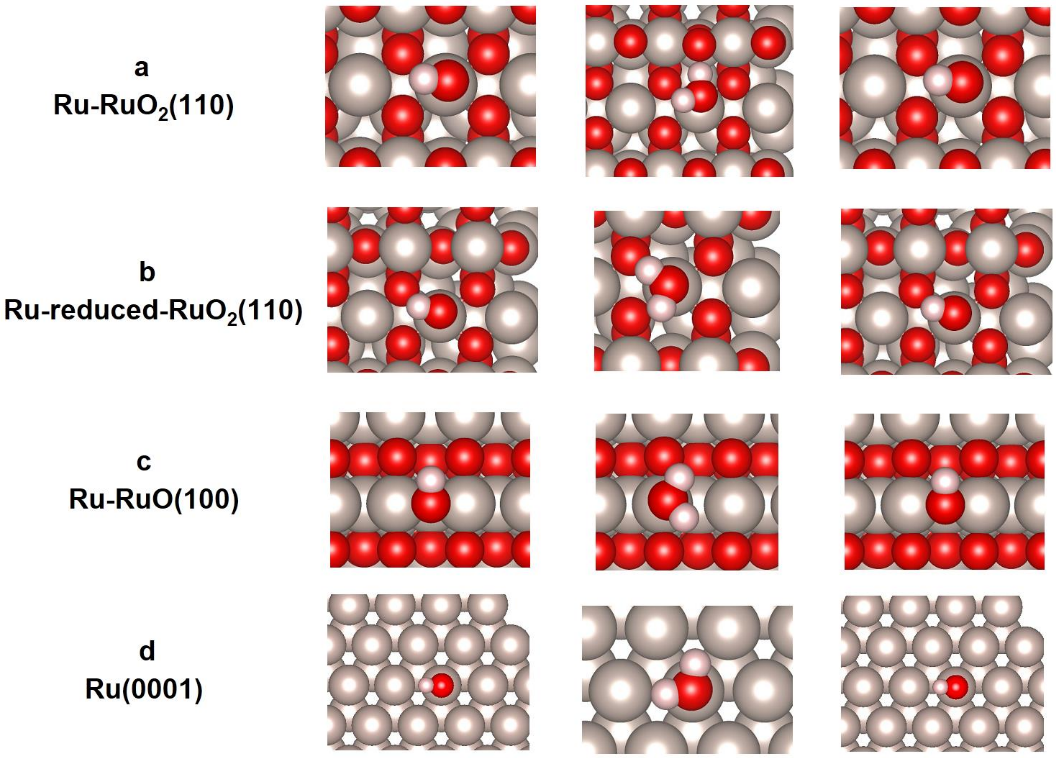

Supplementary Figure 7 | Configurations of reaction intermediates on the reaction pathways of the hydrogen evolution. Ru: gray balls; O: red balls; H: white balls.

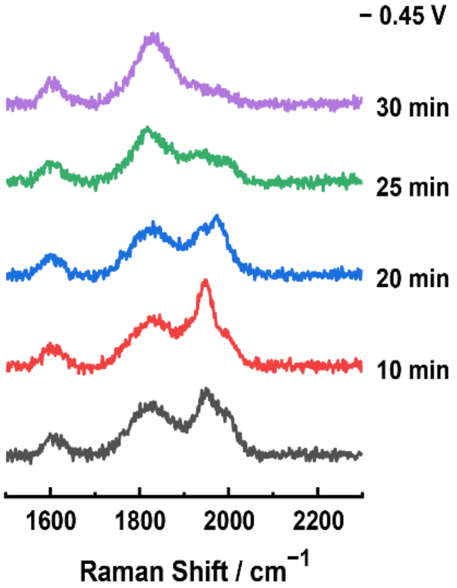

Supplementary Figure 8 | In situ Raman spectra of the distinct *H varying with time at −0.45 V for 55 nm Au@2.5 nm Ru NPs in a 0.1 M NaOH solution saturated with Ar.

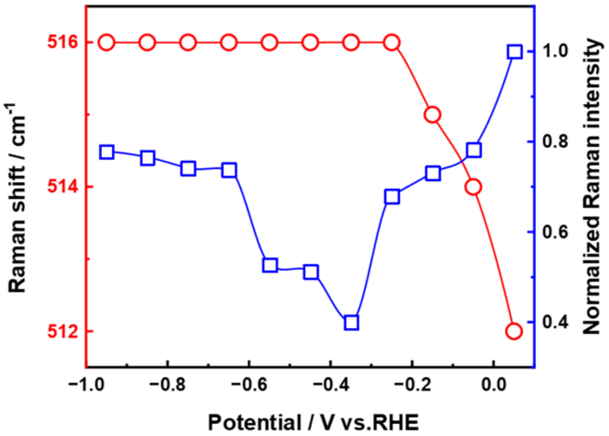

Supplementary Figure 9 | Normalized Raman intensities (blue) and frequency shifts (red) of the Ru-O band at Ru surfaces in the HER potential range. The solution is 0.1 M NaOH saturated with Ar.

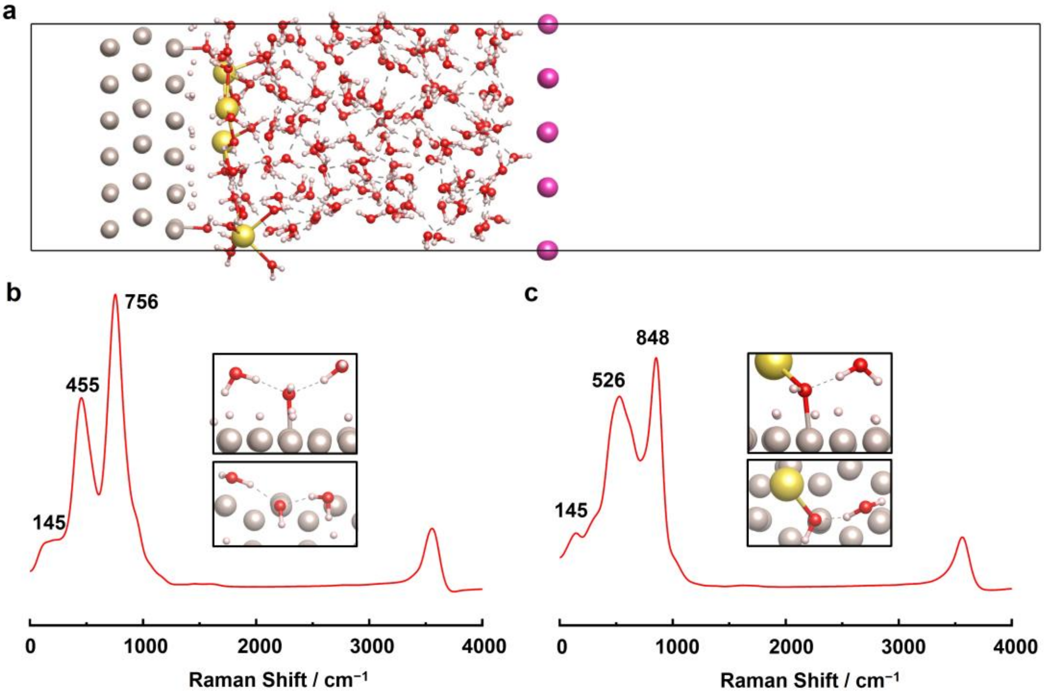

Supplementary Figure 10 | (**a**) Model of Ru(0001)-H_{ad}/water interface at −0.96V vs. SHE. The coverage of *H on Ru surfaces is 2/3 M, and two *OH are adsorbed. Ru: gray; Ne: purple; Na: yellow; O: red; H; white. (**b**) Vibrational density of states of *OH hydrogen bonding with water. (**c**) Vibrational density of states of *OH hydrogen bonding with Na.

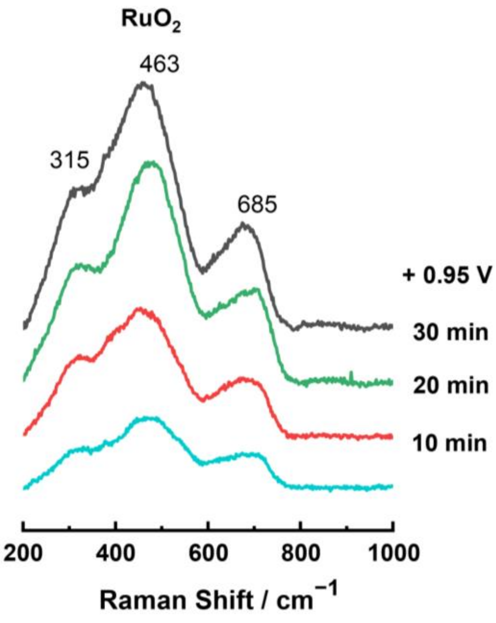

Supplementary Figure 11 | The time-course change of the RuO_2 Raman peak intensity of Au@Ru NPs in a 0.1 M NaOH solution at +0.95 V.

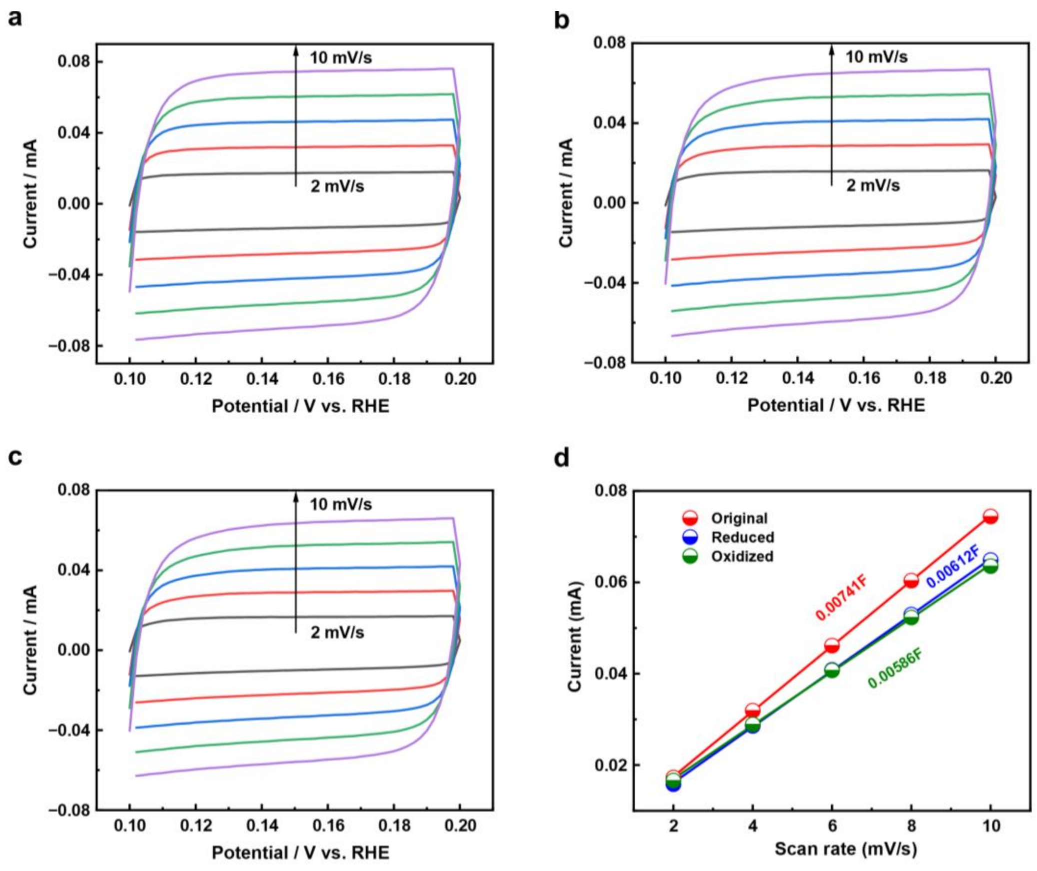

Supplementary Figure 12 | Electrochemical active surface area (ECSA). Cyclic voltammograms of different valence Ru in a non-faradaic region at different scan rates, (**a**) Original, (**b**) Reduced, (**c**) Oxidized. (**d**) Scan rate dependence of the current at 0.15 V vs. RHE.

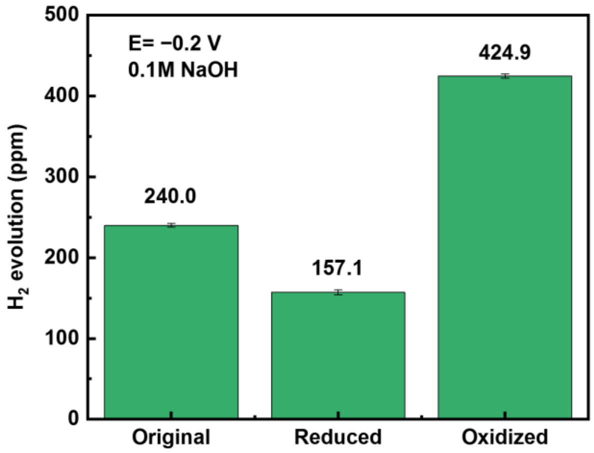

Supplementary Figure 13 | The hydrogen production for HER at −0.2 V of Au@Ru with different valence Ru.

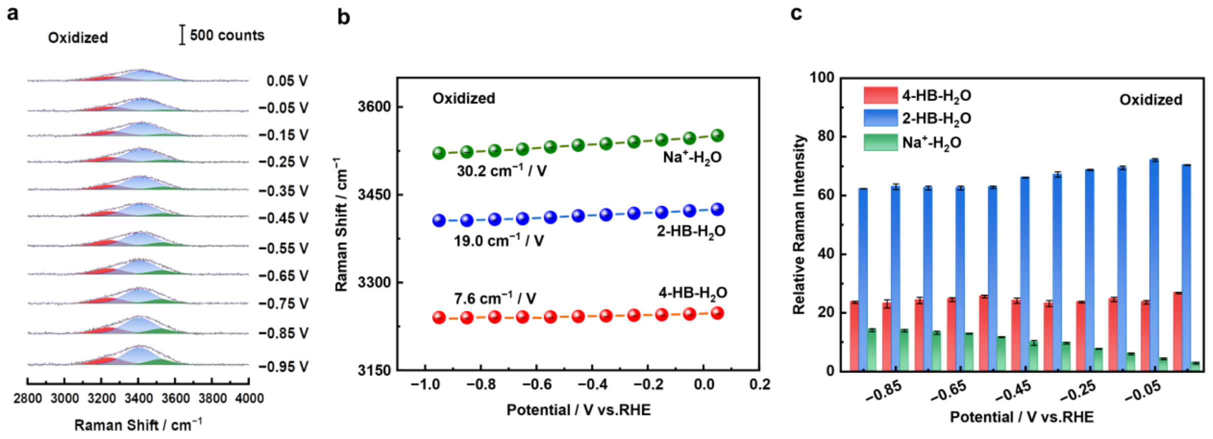

Supplementary Figure 14 | (**a**) In situ enhanced Raman spectra of the interfacial water on the Au@Ru surface (Oxidized) in 0.1M NaOH. Gaussian 13fits of three O-H stretching modes are shown in red, blue, and green, respectively. (**b**) Frequency plot of changes in the O-H stretching modes in Raman spectra of interfacial water from Supplementary Figure 7a. (**c**) Trend chart of Relative Raman Intensity about three O-H stretching modes of interfacial water on the Au@Ru surface (Oxidized) in 0.1M NaOH.

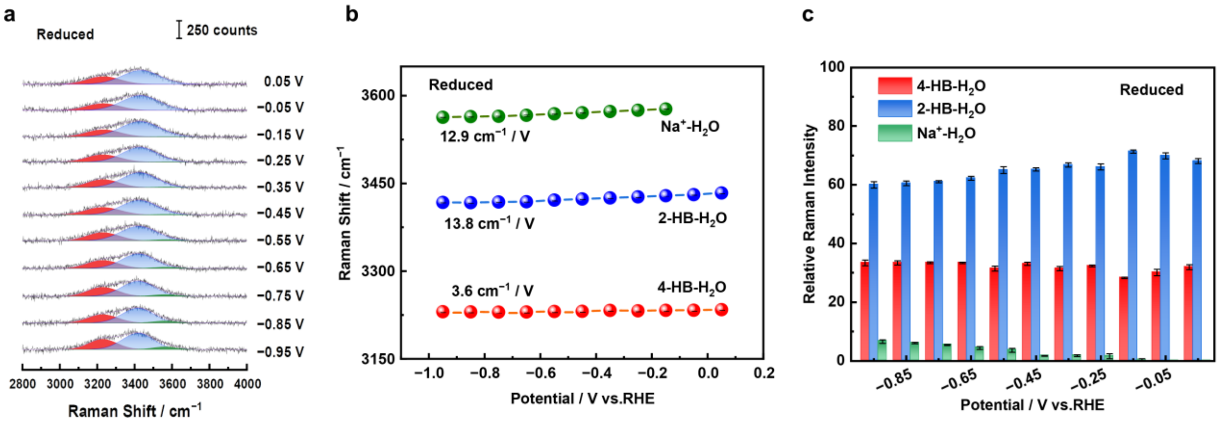

Supplementary Figure 15 | (**a**) In situ enhanced Raman spectra of the interfacial water on the Au@Ru surface (Reduced) in 0.1M NaOH. Gaussian fits of three O-H stretching modes are shown in red, blue, and green, respectively. (**b**) Frequency plot of changes in the O-H stretching modes in Raman spectra of interfacial water from Supplementary Figure 8a. (**c**) Trend chart of Relative Raman Intensity about three O-H stretching modes of interfacial water on the Au@Ru surface (Reduced) in 0.1M NaOH.

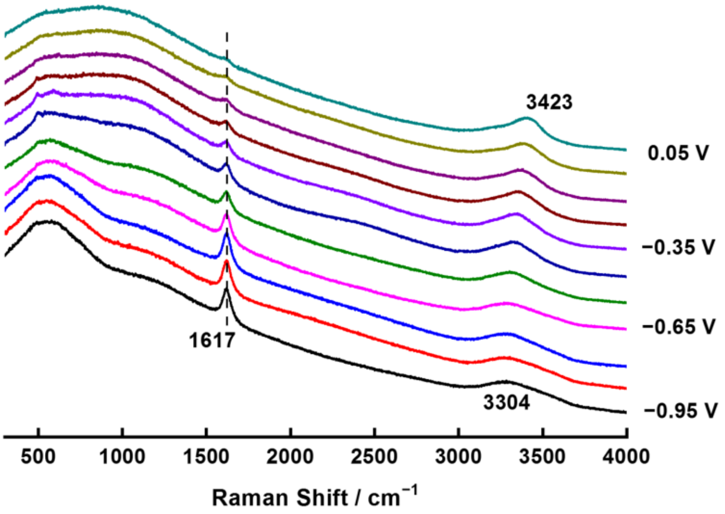

Supplementary Figure 16 | In situ enhanced Raman spectra of the HER process on Au NPs surface in a 0.1 M NaOH solution.

Supplementary Table 1. The vibrational frequencies of Ru-H stretching, Ru-OH bending, Ru-O rotation, and Ru-O stretching modes on different surfaces.

Surfaces	Species	Adsorption site	Ru-H stretching (cm^{-1})	Ru-OH bending (cm^{-1})	Ru-O rotation (cm^{-1})
Ru-RuO_2(110)	H	5c-Ru	1987		
	O	5c-Ru			527
Ru-reduced-RuO_2(110)	H	5c-Ru	1991		
Ru-RuO(100)	H	Top-Ru	1947		
Ru(0001)	H	Top-Ru	1854		
	OH	Top		752	
	OH (AIMD)	Top		756	

Supplementary Table 2. The Bond length of Ru-H, Mulliken charge of *H, and work functions of different Ru surfaces.

	D_{Ru-H}	Mulliken charge of *H	Work function(eV)
RuO_2(110)	1.615	0.009578	5.40
Reduced-RuO_2(110)	1.604	0.007181	
RuO(100)	1.609	0.026265	
Ru(0001)	1.639	0.047717	5.04

Supplementary Table 3. ICP-AES results of Au@Ru

	Au(ppm)	Ru(ppm)
Au@Ru	21.565	1.688

Supplementary Table 4. The zero point energies (ZPE) and correction terms of solvation energies of the surface adsorbed species.

Surfaces	Species	Adsorption site	ZPE (eV)	Solvation energy (eV)
Ru-RuO_2(110)	H	5c-Ru	0.2	−0.07
	OH	5c-Ru	0.35	−0.54
	H_2O	5c-Ru	0.71	−0.36
Ru-reduced-RuO_2(110)	H	5c-Ru	0.2	−0.07
	OH	5c-Ru	0.35	−0.54
	H_2O	5c-Ru	0.71	−0.36
Ru-RuO(100)	H	Bridge-Ru	0.2	−0.12
	OH	Bridge-Ru	0.37	−0.38
	H_2O	Top-Ru	0.71	−0.36
Ru(0001)	H	Hollow	0.17	−0.12
	OH	Top	0.35	−0.54
	H_2O	Top	0.71	−0.36

Supplementary References

1. Goyal, A., & Koper, M. T. The interrelated effect of cations and electrolyte pH on the hydrogen evolution reaction on gold electrodes in alkaline media. *Angew. Chem., Int. Ed.* **60**, 13452-13462 (2021).
2. Li, C. Y. et al. In situ monitoring of electrooxidation processes at gold single crystal surfaces using shell-isolated nanoparticle-enhanced Raman spectroscopy. *J. Am. Chem. Soc.* **137**, 7648-7651 (2015).
3. Goyal, A., & Koper, M. T. M. The interrelated effect of cations and electrolyte pH on the hydrogen evolution reaction on gold electrodes in alkaline media. *Angew. Chem. Int. Ed.* **60**, 13452-13462 (2021).
4. Juodkazytė, J., Vilkauskaitė, R., Šebeka, B., & Juodkazis, K. Difference between surface electrochemistry of ruthenium and RuO_2 electrodes. *Trans. IMF* **85**, 194-201 (2007).
5. Dória, A. R. Influence of the RuO_2 layer thickness on the physical and electrochemical properties of anodes synthesized by the ionic liquid method. *Electrochim. Acta* **354**, 136625 (2020).
6. Cong, N. et al. Nanoporous RuO_2 characterized by RuO $(OH)_2$ surface phase as an efficient bifunctional catalyst for overall water splitting in alkaline solution. *J. Electroanal. Chem.* **881**, 114955 (2021).
7. She, C. X. et al. The investigation of electro-oxidation of methanol on Pt-Ru electrode surfaces by in-situ Raman spectroscopy. *J. Korean Electrochem. Soc.* **5**, 221-225 (2002).
8. Park, S., Wieckowski, A., & Weaver, M. J. Electrochemical infrared characterization of CO domains on ruthenium-decorated platinum nanoparticles. *J. Am. Chem. Soc.* **125**, 2282-2290 (2003).
9. Goyal, A., & Koper, M. T. M. The interrelated effect of cations and electrolyte pH on the hydrogen evolution reaction on gold electrodes in alkaline media. *Angew. Chem. Int. Ed.* **60**, 13452-13462 (2021).
10. Li, J. F., Zhang, Y. J., Ding, S. Y., Panneerselvam, R., & Tian, Z. Q. Core–shell nanoparticle-enhanced Raman spectroscopy. *Chem. Rev.* **117**, 5002-5069 (2017).
11. Su, P. P. et al. Exceptional electrochemical HER performance with enhanced electron transfer between Ru nanoparticles and single atoms dispersed on a carbon substrate. *Angew. Chem., Int. Ed.* **133**, 16180-16186 (2021).
12. Fang, W. C. et al. Arrayed CN_xNT–RuO_2 nanocomposites directly grown on Ti-buffered Si substrate for supercapacitor applications. *Electrochem. Commun.* **9**, 239-244 (2007).
13. Wang, Z. P. et al. In-situ surface decoration of RuO_2 nanoparticles by laser ablation for improved oxygen evolution reaction activity in both acid and alkali solutions. *J. Energy Chem.* **54,** 510-518 (2021).
